# The influence of gluteal muscle strength deficits on dynamic knee valgus: a scoping review

**DOI:** 10.1186/s40634-022-00513-8

**Published:** 2022-08-17

**Authors:** Vito Gaetano Rinaldi, Robert Prill, Sonja Jahnke, Stefano Zaffagnini, Roland Becker

**Affiliations:** 1 II Clinica Ortopedica e Traumatologica, IRCCS Istituto Ortopedico Rizzoli, 40136 Bologna, Italy; 2 Medical School Theodor Fontane, 14770 Brandenburg, Germany; 3 Center of Orthopaedics and Traumatology, University Hospital Brandenburg/Havel, Brandenburg, Germany; 4 Faculty of Health Sciences Brandenburg, Brandenburg Medical School Theodor Fontane, 14770 Brandenburg, Germany; 5 DIBINEM, University of Bologna, Bologna, Italy

**Keywords:** Anterior Cruciate Ligament (ACL), Knee, Biomechanics, Gluteal Muscles, Dynamic Knee Valgus, Muscles Strengths

## Abstract

Anterior cruciate ligament (ACL) injuries are caused by both contact and non-contact injuries. However, it can be claimed that non-contact ones account approximately for 70% of all cases. Thus, several authors have emphasized the role of reduction of muscle strength as a modifiable risk factor referred to non-contact ACL injury, with the latter being targeted by specific training interventions.

The present paper wants to review the available literature specifically on the relationship between dynamic knee valgus, gluteal muscles (GM) strength, apart from the potential correlation regarding ACL injury.

After a research based on MEDLINE via PubMed, Google scholar, and Web of Science, a total of 29 articles were collected and thus included.

Additionally, this review highlights the crucial role of gluteal muscles in maintaining a correct knee position in the coronal plane during different exercises, namely walking, running, jumping and landing.

## Background

Recently, the incidence of anterior cruciate ligament (ACL) injuries has gradually increased [1]. According to a 2021 review article, the incidence rate of ACL injuries is estimated at 36.9 per 100,000 individuals in the general population [2]. Additionally, ACL injuries are caused by both contact and non-contact injuries, but the majority of them (approximately 70%) actually belong to non-contact ones [3]. Here, injuries are sustained without extrinsic contact to the knee, and result from the athlete’s inherent movement patterns [4].

For this, numerous studies have evaluated the etiology of ACL injuries. Intrinsic, non-modifiable factors can be distinguished to extrinsic and modifiable factors [5]. Following the idea of noncontact ACL injuries being theoretically preventable, identification of modifiable risk factors is essential for successful ACL injury prevention programs.

Besides, several authors have emphasized the reduction of muscle strength as a modifiable risk factor for non-contact ACL injury, the latter being targeted by specific training interventions [6–10]. In the recent months and years, thus, some studies focused on the contribution of the gluteal muscles (GM) in preventing knee sprains during common tasks especially during landing after a jump, or side-cutting movement [4, 5, 11, 12]. Indeed, excessive hip adduction and knee valgus position is proposed as a common risk factor for a variety of acute and overuse lower extremity injuries, including the ACL [13]. Eventually, medial knee joint movement during hip adduction might be an explanation for this to happen, causing dynamic valgus and significant knee abduction moments. Furthermore, it has been noted that people who leaned predominantly on the hip muscles for absorbing impact forces during landing showed limited knee valgus angles, abduction moments, and energy absorption at the knee [14]. Several studies have reported weakness issues concerning hip extension, external rotation and abduction in those participants showing valgus during dynamic tasks or going on to suffer knee injuries [7, 14–16].

Hip abduction and external rotation are predominantly positively influenced through both the gluteus medius (GMe) and gluteus maximus muscle (GMa) [8, 17].

In general, GMe and GMa muscles are the key muscles contributing pelvic stability and lower extremity function. They are frequently implicated in disorders of the knee, the pelvis, and the hip [18].

For our purpose, the present scoping review will focus on the gluteus muscle dysfunction on dynamic knee valgus.

## Methods

### Objective

The scoping review method was specifically chosen here so as to lay out a complete summary of the current literature and knowledge, with the explicit hope of spurring subsequent studies and articles in this field [19]. Moreover, the review was conducted according to the recommendations by Peters et al. [20] and based on the Preferred Reporting Items for Systematic reviews and Meta-Analyses extension for Scoping Reviews (PRISMA-ScR) [4]. The PRISMA checklist can be found in the supplements.

This scoping review aims at overviewing the available literature on the relationship between dynamic knee valgus, GM-strength and the potential correlation regarding ACL injury. It is hypothesized that evidence supports the idea of solid GM strength being highly associated with few dynamic knee valgus (DKV). Subsequently, relations between GM strength and ACL injury will be reported.

### Concept and context

Our main focus of the scoping review was to extract information about the association between GM strength and DKV. Therefore, the phenomena of interest in GM are expressions of muscle strength, namely isokinetic or isometric abduction, Electromyography (EMG) GM voluntary contraction, or initial activation during landing tasks. In terms of knee position, the maximum valgus - or so-called abduction of the knee - will be included, in this case measured with a sensor camera. Both issues must be addressed in the same study so as to enable comparisons.

### Inclusion criteria

All studies reporting the association of performance-based assessed knee valgus and gluteal muscles, were included. In addition, studies which have previously examined healthy subjects and participants with knee injuries were also included. For information completeness, studies were eligible for inclusion provided they have been published in English, Italian or German languages.

### Search strategy

The search strategy aims at presenting a comprehensive overview of the existing research. Therefore, published data from all available study designs was searched with a title and with abstract search strategy, where the latter was deemed to be possible. The databases MEDLINE via PubMed, Google scholar, and Web of Science were employed for the research. After a preliminary search of the literature the search terms were finally defined for the analysis of the entire literature. Last, the search was performed during the month of December 2021.

## Results

**Fig. 1:**
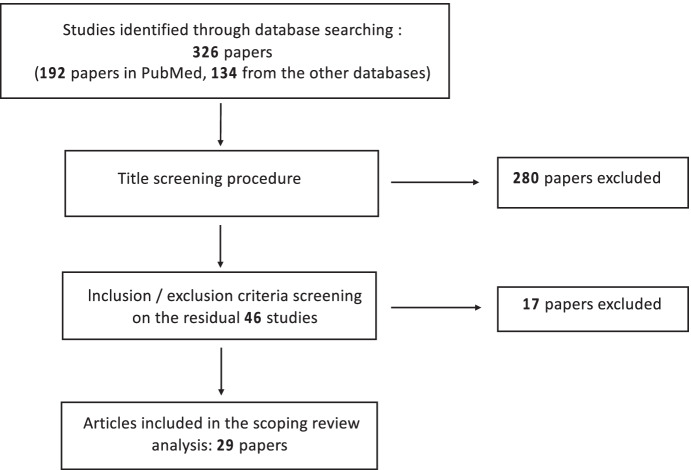
Flow diagram of the study selection

Our search strategy identified 326 results and 192 papers in PubMed: Following a duplication removal, another 134 result from the other databases were added. Moreover, after a title screening procedure, 280 articles were excluded from the pool. The remaining 46 articles were further examined using the inclusion/exclusion criteria with the exclusion of 17 sources (Fig. 1). At last, twenty-nine papers were finally included for our purposes.

The study characteristics, level of evidence, outcomes, setting, methods, and results of the included studies were summarized in Table 1.

**Table 1:** The study characteristics, level of evidence, outcomes, setting, methods, and results of the included studies

***JUMP-LANDING TASKS***						
*Study*	*Study Design, Level of Evidence*	*Number of partecipants,age, sex*	*Outcome Parameters*	*Setting*	*Method*	*Results*
*Ueno* et al. *2020* [4, 17]	*Descriptive laboratory study,Level of evidence III*	*30 females (mean age 15.6 ± 1.6 years)*	*relationships between knee abduction moment and frontal plane biomechanics*	*Motion analysis and electromyography*	*Jump-landing task from a 30 cm step*	*Knee abduction moment was predicted by lower gluteus medius strength*
*Llurda-Almuzara* et al. *.2020* [21]	*Descriptive laboratory study, Level of evidence III*	*50 healthy men (between 18 to 29 years old)*	*Dynamic knee valgus (DKV) and hip and knee neuromuscular response (NMR).*	*Motion analysis and electromyography*	*Jump-landing task from a 50 cm step*	*No correlation between DKV and NMR and this could be explained because of the influence of Central Nervous System*
*Cronin* et al. *2016* [22]	*Descriptive laboratory study, Level of evidence III*	*40 healthy active females (mean age, 21.0 ± 1.7 years)*	*Relationship between Hip abduction and knee motion during a single-leg jump-cutting task in females.*	*Motion analysis and electromyography*	*Single-leg jump-cuts*	*The gluteus maximus, functioning as a hip abductor may take on a pivotal role in controlling hip adduction and knee valgus motion during these types of tasks*
*Smeets* et al. *2019 *[23]	*Randomized controlled trial, Level of evidence I*	*21 athletes who had an ACLR and 21 uninjured controls (mean age 23.8 ± 4.2 years and* 21.5 ± 1.5 years)	*Comparison between ACLR athletes and uninjured athletes during landing kinematic, kinetic and/or muscle activation profiles fatigue induced.*	*Motion analysis and electromyography*	*Single leg hop,medial and lateral hop,vertical hop with 90° of medial or lateral rotation*	*ACLR athletes and uninjured athletes have similar biomechanical and neuromuscular responses to fatigue.*
*Neamatallah* et al. *2019 *[39]	*Correlation study, Level of evidence III*	*17 males and 17 females respectively aged 26.9 ± 3.8 years and 25.7 ± 4.5 years*	*Gluteal muscle EMG activity related to hip abduction and extension muscle strength and consequent knee and hip angles and moments.*	*Motion analysis and electromyography*	*Single-leg Squat,Forward Land and Side land) from a 30 cm platform.*	*In females knee abduction moments and angles were strongly correlated to hip abduction strength. In males the relationships were less clear.*
*Patrek* et al.*, 2011* [10]	*Descriptive laboratory study Level of evidence IV*	*20 physically active women (mena age 21.0 ± 1.3 years)*	*examine the changes in single-leg landing mechanics and gluteus medius recruitment that occur after a hip-abductor fatigue protocol.*	*Maximum isometric strength of the hip abductors was tested using a Nicholas MMT handheld dynamometer and stabilization strap*	*Participants were tested before and after a hip-abductor fatigue protocol.*	*Changes observed during single-leg landings after hip-abductor fatigue were not generally considered unfavorable to the integrity of the anterior cruciate ligament.*
*Study*	*Study Design, Level of Evidence*	*Number of partecipants,age,sex*	*Outcome Parameters*	*Setting*	*Method*	*Results*
*Hogg* et al.*, 2021* [24]	*Comparative study, Level of evidence III*	*45 healthy females (mean age 20.1 ± 1.7); and 45 healthy males (mean age 20.8 ± 2.0)*	*Gluteal strength and activation mediate associations between femoral alignment measures and functional valgus collapse.*	*Three-dimensional biomechanics and surface electromyography.*	*Single-leg forward landing.*	*In females less gluteal strength and higher muscle activation were marginally associated with valgus movement. In males, less gluteal strength was associated with a more varus posture.*
*Dai* et al.*, 2014* [25]	*Comparative study, Level of evidence III*	*13 male and 15 female recreational athletes (mean age: 21.1¬ ± 2.4 years)*	*Quantify the effects of a resistance band on internal hip abduction moments and gluteus medius activation during the pre-landing and early-landing phases of a jump–landing–jump task.*	*Surface electrodes, retroreflective markers. Reaction forces were collected using a force plate.*	*jump-landing-jump tasks with or without a resistance band applied to their lower shanks.*	*A resistance band applied to the lower shanks increased internal hip abduction moments and gluteus medius EMG during the pre-landing and early-landing phases of a jump–landing and jump task.*
*Homan* et al. *2012 *[8]	*Comparative study, Level of evidence III*	*41 males, 41 females aged respectively 21 ± 3 and 21 ± 3 years*	*Define the Influence of hip strength on gluteal activation and knee valgus motion.*	*Electromyography*	*Maximal isometric hip abduction and external rotation contractions during jump landing.*	*Weaker individuals compensate for a lack of force production* via *heightened neural drive. As such, hip muscle strength influences knee valgus motion indirectly by determining neural drive requirements*
*Rath* et al.*, 2016* [26]	*Descriptive laboratory study Level of evidence IV*	*12 female soccer players (mean age: 19.4 ± 1.4 years, with supple planus (SP) or rigid feet (RF).*	*Determine the degree to which subtalar joint pronation resulting from a SP foot affects knee alignment, hip muscle activation and ground reaction force attenuation*	*Surface EMG and force plate*	*three broad jump-to-cut trials*	*Decreased hip muscle activation during a broad jump-to-cut maneuver is associated with SP foot. This result in increased risk of non-contact ACL injury in female soccer players.*
*Sinsurin* et al.*, 2020* [27]	*Descriptive laboratory study Level of evidence IV*	*12 Males and 8 Females (mean age 30.8 ± 7 years)*	*Define differences in the trunk, pelvis,hip, and knee joints, and gluteus medius muscle activity*	*Motion analysis and electromyography*	*Walking and step down from two riser heights.*	*Gluteus Medius has a greater stabilizing role during the step-down tasks*
*Lubahn* et al.*, 2011* [28]	*Descriptive laboratory study, Level of evidence III*	*18 healthy females (mean age 22.3 ± 2.3 years)*	*Examine the muscular activation of the gluteus maximus and gluteus medius*	*Motion analysis and electromyography*	*Double and single leg squat*	*The SLS was most effective exercise for activating the gluteus maximus and gluteus medius. Applied knee load does not appear to increase muscle activation during SLS and FSU*
*Study*	*Study Design, Level of Evidence*	*Number of partecipants,age, sex*	*Outcome Parameters*	*Setting*	*Method*	*Results*
*Russell* et al.*, 2006* [29]	*Comparative study, Level of evidence III*	16 Males mean age 24.6 ± 5 years, and 16 females: mean age 21.6 ± 6 years)	*Determine if frontal-plane knee angle and Gluteus medius activation differ between the sexes at initial contact and maximal knee flexion during a single-leg drop landing.*	*Motion analysis laboratory*	*Evaluation at initial contact and maximal knee flexion during a single-leg drop landing.*	*Women tended to land in more knee valgus before and at impact than men. The GM muscle activation did not differ between the sexes.*
*Hollman* et al. *2013 *[30]	*Descriptive laboratory study Level of evidence IV*	40 *Active, healthy women age*d *18 to 36*	*Determine if hip muscles are correlated to frontal plane knee motion.*	*handheld dynamometer, electromyography*	*jump-landing task*	*Hip-extensor strength and gluteus maximus recruitment are both associated with frontal-plane knee motions during a dynamic weight-bearing task.*
*Cannon* et al. *2019* [6]	*Descriptive laboratory study Level of evidence IV*	18 Female participants (mean age: 20.7 ± 1.3 years)	*Investigate the influence of lumbar spine joint rotational stiffness (JRS), and gluteal musculature contribution to hip JRS, on dynamic knee valgus*	*Electromyography*	*Drop vertical jump*	*Increased JRS at the lumbar spine and greater JRS contributions from the gluteal musculature are linked with preventing high medial knee displacement.*
*Camparis Lessi* et al.*, 216*	*Comparative study, Level of evidence III*	7 Men mean age 23.90 ± 2.80 and 7 women mean age 24.7 ± 5.3 years)	*Compare the effects of muscle fatigue on trunk, pelvis and lower limb kinematics and on lower limb muscle activation between male and female athletes who underwent ACL reconstruction.*	*Laboratory setting*	*Single leg drop vertical jump task, before and after a lower limb muscle fatigue protocol*	*Muscle fatigue produced kinematic alterations that have been shown to increase the risk for a second ACL injury in female athletes.*
*Fadari Dehcheshmeh* et al. *2021* [31]	*Descriptive laboratory study Level of Evidence IV*	34 professional female athletes aged 18.29 ± 3.29 years	*Influence of Lumbo-Pelvic Control (LPC) on landing mechanics and lower limb muscle activity in professional athletes engaged in sport requiring frequent landing.*	*Electromyography was used to record the activity of the gluteus medius (GMed), rectus femoris, and semitendinosus.*	*Jump-landing tasks*	*Poor lumbopelvic control afects the kinematics and activity of the lower limb muscles, and may be a risk factor for lower limb injuries, especially of the knee*
***GLUTEAL FATIGUE DURING RUNNING***						
*Study*	*Study Design, Level of Evidence*	*Number of partecipants,age, sex*	*Outcome Parameters*	*Setting*	*Method*	*Results*
*Baker* et al. *2018* [28]	*Comparative study, Level of evidence III*	16 Men mean age 33.70 ± 7.80 and 14 women mean age 32.7 ± 6.3 years)	*Influence of Iliotibial Band Syndrome in Knee and Hip Adduction and Hip Muscle activation in runners.*	*Motion capture, Surface electromyography*	*30 minutes running evaluation*	*Injured runners demonstrated increased knee adduction compared with control runners at 30 minutes*
*Willson* et al. *2012* [12]	*Comparative study, Level of evidence III*	19 Men mean age 20.4 ± 1.80 and 19 Women mean age 20.5 ± 1.5 years)	*Differences in the timing and magnitude of gluteal muscle activity as well as hip and knee joint frontal and transverse plane kinematics between male and female runners in the context of this gender bias.*	*Motion analysis and electromyography*	*Running evaluation*	*Females ran with 40% greater peak gluteus maximus activation level and 53% greater average activation level than males. Female runners also displayed greater hip adduction and knee abduction angles at initial contact.*
***GLUTEAL RESPONSE DURING WALKING***						
*Sritharan* et al. *2012* [32]	*Descriptive laboratory study Level of Evidence IV*	*8 healthy males (age: 26 ± 4 years)*	*Muscle contribution to the external knee adduction moment, tibiofemoral joint reaction force, and reaction moment.*	*Motion analysis and electromyography*	*Walking*	*Gluteus medius contributed substantially to the medial compartment compressive force leading to greater dynamic knee valgus*
*Pohl* et al. *2015* [33]	*Descriptive laboratory study Level of Evidence IV*	*8 healthy, active males (mean age 27 ± 6 years)*	*To experimentally reduce hip-abduction strength and observe the subsequent changes in frontal-plane biomechanics.*	*Superior gluteal nerve block injection and subsequent gait analysis*	*Walking*	*Reduction in hip-abductor strength was not associated with alterations in the frontal-plane gait biomechanics of young, healthy men*
*Sinsurin* et al. *2019 *[27]	*Descriptive laboratory study Level of Evidence IV*	12 Males and 8 females (mean age 30.9 ± 7 years)	*explore differences in the coronal biomechanics of the trunk, pelvis,hip, and knee joints, and gluteus medius muscle activity during walking and step down*	*Motion analysis and electromyography*	*Walking and step down*	*Significantly greater knee adduction moments were seen during both step-down tasks comparedto level walking with significantly greater Gluteus medius activity*
*DeJong* et al. *2019* [34]	*Descriptive laboratory study Level of Evidence IV*	8 Males and 20 females (mean age 19.6 ± 1.5 years)	Determine differences in Ultrasound Imaging gluteal muscle activity during gait.	*Ultrasound of the gluteus maximus and medius during quiet stance, heel strike, and walking.*	*Walking and quiet stance*	*USI highlighted gluteal activity differences of Medial Knee Displacement limbs during gait, which may contribute to inadequate hip stabilization during this daily repetitive task*
*Henriksen* et al. *2009* [35]	*Descriptive laboratory study Level of Evidence IV*	6 males and 9 females (mean age. 24.8 ± 6.1 years)	Test that reduced function of the gluteus medius muscle would lead to increased external knee adduction moment during walking in healthy subjects.	*Intramuscular hypertonic saline injection and subsequent gait analysis with electromyography*	*Walking*	*Experimentally reduced Gluteus Medius function, leads to reduced internal hip abductor moments and external knee adduction moments.*
***SQUAT TASKS***						
*Study*	*Study Design, Level of Evidence*	*Number of partecipants,age, sex*	*Outcome Parameters*	*Setting*	*Method*	*Results*
*Hollman* et al. *2013 *[30]	*Descriptive laboratory study Level of evidence IV*	40 *Active, healthy women age*d *18 to 36*	*Examine relationships between hip muscle strength, recruitment and frontal plane knee kinematics and knee valgus during squat task*	*Muscle strength was measured bilaterally with a handheld dynamometer, recruitment was measured with surface electromyography.*	*Single leg Squat*	*Gluteus maximus recruitment may modulate frontal plane knee kinematics during single-leg squats..*
*Kim* et al. *2015 *[36]	*Descriptive laboratory study Level of evidence IV*	20 females (mean age 22.6 ± 2.5 years)	*Examine the relationship of anticipatory activity of the gluteus medius to pelvic drop and knee abduction moment.*	*Motion analysis and electromyography*	*Squat task*	*The amount of gluteus medius activity is more important for controlling knee and pelvic stability in the frontal plane than the onset of activation.*
*Nakagawa* et al. *2012* [31]	*Comparative study, Level of evidence III*	20 men with PFPS 20 men without, 20 women with PFPS and 20 without, aged from 18 to 35 years	*differences between sexes in trunk, pelvis,hip, and knee kinematics, hip strength, and gluteal muscle activation during the performance of a single-leg squat in individuals with patellofemoral pain syndrome (PFPS) and control participants.*	*Hip abduction and external rotation eccentric strength was measured on an isokinetic dynamometer*	*Single leg Squat*	*Both males and females with PFPS had reduced eccentric strength of the hip abductors and external rotators.*
*Lubahn* et al. *2011* [28]	*Level of Evidence III*	18 healthy females (mean age 22.3 ± 2.3)	*Examine the muscular activation of the gluteus maximus and gluteus medius during squat task*	*Motion analysis and electromyography*	*Single and double leg squat*	*Single leg squat achieved the highest activation of the gluteus maximus and gluteus medius*
*Padua* et al. *2012* [37]	*Descriptive laboratory study* *Level of evidence IV*	60 partecipants,30 females (23.9 ± 3.6) and 30 males 22.2 ± 2.6.	*Compare hip muscle strength in patients with Knee Medial Displacement*	*Motion analysis and electromyography*	*Double leg squat*	*Medial knee displacement during squatting tasks appears to be associated with increased hip-adductor activation.*

### Gluteal evaluation during jumping and landing tasks

Seventeen studies evaluated the GM influence on the DKV during a landing and jumping task [6, 8–10, 17, 21, 22, 24–30, 32, 38, 39]. Ten were descriptive laboratory studies [6, 10, 17, 21, 22, 26–28, 30, 38], while six were comparative studies that compare frontal-plane knee angle and GMe activation between the sexes during a jump-landing test [8, 9, 24, 25, 29, 39]. Additionally, one was a randomized controlled trial (RCT) [32].

The descriptive laboratory studies were conducted with video analysis compared to GM activation using EMG.

Seven studies have underlined the important stabilizing role of GM strength during the jump-landing task, including the prevention DKV deviation [10, 17, 22, 27, 28, 30, 39]: However, only one study found no correlation between knee valgus and gluteal muscle strength [21]. Thus, these studies highlight the importance of GMa and GMe. The comparative studies were conducted to demonstrate how GM strength and activation are sex correlated, as well as how abduction moment can actually influence knee injuries in women and men.

In the RCT, the authors compare GM activity in athletes after ACL reconstruction and uninjured athletes during landing kinematic, discovering that they have similar biomechanical and neuromuscular responses.

### Gluteal fatigue during running

Two studies have investigated gluteal muscle fatigue during a running session [12, 40]. First, one study compared runners with iliotibial band syndrome as opposed to healthy runners [40]. The authors concluded that injured runners demonstrated increased knee adduction in comparison to controls after just a 30 minutes run.

The second study [19] besides evaluated the differences in the timing and magnitude of gluteal muscle activity between male and female runners. Female athletes were observed to run with a 40% greater peak gluteus maximus activation level, and a 53% greater mean activation level, when compared to males. Additionally, female runners also showed greater hip adduction and knee abduction angles during initial ground contact.

### Gluteal response during walking

Evidence from five studies concerning the GM response during walking sessions was evaluated [27, 33, 35, 41, 42]. Four of them analyzed gluteus activity through (EMG) and motion analysis [27, 32, 33, 35].

The importance of the GMe and GMa for maintaining proper knee alignment in the frontal plane was assessed in three different studies [27, 32, 35]; nevertheless, only one highlighted no difference between gluteal fatigue and knee malalignment [33].

The fifth study, assessing differences in GM activity during gait using ultrasound imaging [41], highlighted that gluteal activity differences lead to medial knee displacement during gait, concluding potential contribution to inadequate hip stabilization during this daily repetitive task.

### Studies that analyze squat tasks

Five studies have here assessed the GMa and GMe strength during the squat task [28, 30, 31, 36, 37].

Of these, three evaluated relationships between hip muscle strength, recruitment, frontal plane knee kinematics, and knee valgus during a squat task [28, 30, 37]. GMa recruitment might modulate frontal plane knee kinematics during single-leg squats.

Moreover, one study examined differences between sexes in knee kinematic and GM activation during a single-leg squat in individuals with patellofemoral pain syndrome (PFPS) and control participants, pointing out that both males and females with PFPS have a reduction in eccentric strength of the hip abductors and external rotators, resulting in a higher DKV [31].

The previous study measured the muscular activation of the GMa and GMe during the squat task, showing the highest activation of these muscles during single-leg squat exercise [36].

**Fig. 2:**
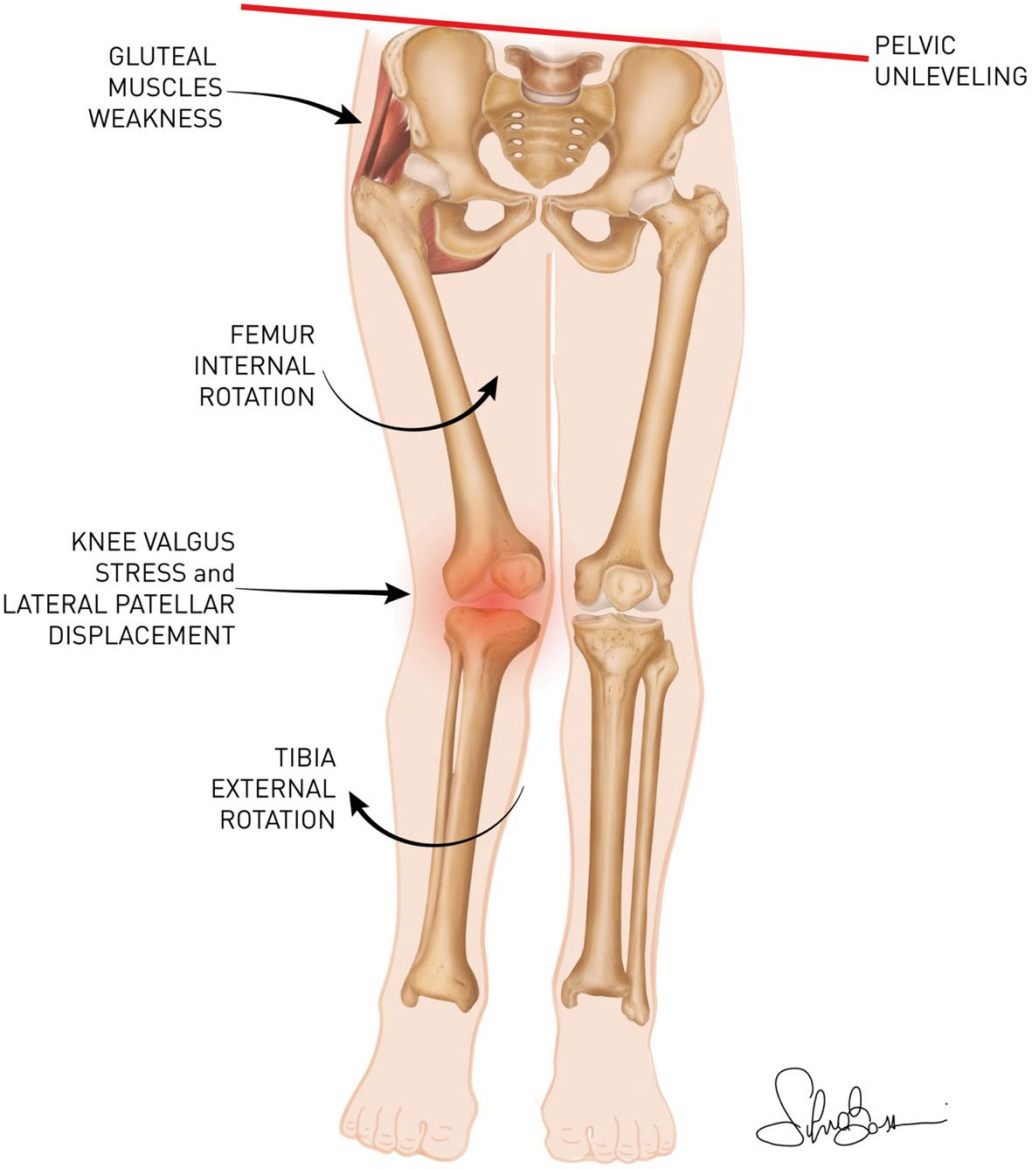
Gluteal Muscles weakness leads to adduction and internal rotation of the femur, valgus at the knee, and tibia external rotation

The influence of gluteal muscles strengths on lower limb biomechanics is summarized in Fig. 2.

## Discussion

Our research crucially highlights the role of the gluteal muscles in maintaining a correct knee position in the coronal plane during different exercises, namely walking, running, jumping and landing.

Besides, numerous studies have shown that greater strength of the GMe and GMa may prevent DKV and thus non-contact ACL injuries.

In addition, several authors demonstrated how hip abduction moments influence DKV during jump landing task [5, 9–12, 15, 16, 34].

In particular, it has been proven that increased knee abduction moment is predicted by reduced GMe force, causing increased lateral ground reaction forces during a jump in both young males and females [16, 21, 27, 39, 42].

The analysis of forty recreationally active females has shown that the GMa works partially as a hip abductor and plays a pivotal role in controlling hip adduction and knee valgus motion during a landing task [22]. Women tend to land in a more knee valgus position than men [29]. However, in contrast to the GMa, the GMe activation did not differ between the sexes, providing thus a possible explanation of anterior cruciate ligament injuries in terms of sex disparity. Several laboratory studies have proven neuromuscular deficits and muscular fatigue, causing knee kinematic alterations and the increased risk for ACL injury in female athletes [6, 9]. Here, fatigue enhanced landing deficits in ACL reconstructed knee in athletes during a landing task [32]. However, fatigue did not affect knee abduction moments in the uninjured leg and control group.

On top of this, we identified six studies that explored the correlation between coronal biomechanics knee joint displacement and GMe activity during walking or running [27, 33, 35, 40, 41, 43].

The majority of these included researches demonstrating the crucial influence of the gluteal muscles on maintaining a correct coronal axis of the knee during activities, such as walking or running [27, 40, 43].

In particular, an Ultrasound Imaging (USI) study on gluteal activity during gait highlighted the close correlation between Medial Knee Displacement (MKD), GM recruitment, and muscle belly change during walking, concluding that the GMe significantly contributes to frontal limb stabilization during this daily repetitive task [41].

An experimental study showed that reduced GM function caused by intramuscular hypertonic saline injections leads to reduced internal hip abductor moments and external knee adduction moments [35].

These findings have proven, once again, the close correlation between gluteal muscle strength and DKV.

In contrast, only one single study could not find an explicit relation between GMe strength and frontal plane knee deviation [33]. Furthermore, the authors found no alterations in hip-adduction moment, hip adduction, or contralateral pelvic drop following a reduction in hip-abductor strength after a transient gluteal nerve block.

In addition, various investigations have analyzed the influence of the GM on valgus knee control during a squatting task [28, 30, 36, 37, 44]. In particular, it has been showed the anticipatory activity of the GM to pelvic drop and knee abduction moment on a total of sixty-one healthy females examined. The latter study has claimed that GMa and GMe recruitment may modulate frontal plane knee kinematics during single-leg squats [30, 36].

Furthermore, a laboratory comparative study has proven that the single-leg squat achieved the highest integrated and peak activation of the GMa and GMe [28].

On top of these papers, Nakagawa et al. [31] have found increased hip internal rotation and decreased GMe activation during a single-leg squat in females with patellofemoral pain syndrome (PFPS) compared to female control participants. This conclusion strengthens the hypothesis that altered knee kinematics hurts the other muscle groups and vice versa.

Although reviewed published literature has proven to be highly insightful, key methodological and research design limitations must be addressed so as to move forward, so as to enable and improve our understanding of effective gluteal muscle influence on knee sprain injuries.

These limitations include small sample sizes, lack of control group, and only partly comparable GM study methods such as video analysis, EMG, strength evaluation with a handheld dynamometer, and Ultrasound evaluation.

Moreover, most of the available studies are descriptive, with a level of evidence IV.

For this reason, especially prospective and cross-sectional studies with injured and control groups are needed to investigate through randomized controlled trial studies the influence of the gluteal muscles on DKV. Here, a broadly accepted core measurement set being respected by involved research groups could help harmonize research on the role of gluteal muscles for injury prevention.

 Table 2 is a summary of the clinical implication of the present study.

**Table 2:** Clinical implications

Key finding from the review	Clinical implications
Gluteal muscles strength prevents Dynamic knee valgus	
Gluteal muscles influence the coronal axis of the knee during activities such as walking, running, and squat tasks.	Altered knee kinematics hurts the other muscle groups and vice versa.
Patients with patellofemoral pain syndrome show increased hip internal rotation and decreased Gluteal Muscle activation during a single-leg squat.	A strong gluteal musculature would be able to prevent knee sprain injuries

## Data Availability

The datasets used and analysed during the current study are available from the corresponding author.

## References

[1] Siegel L, Vandenakker-Albanese C, Siegel D (2012). Anterior cruciate ligament injuries: anatomy, physiology, biomechanics, and management. Clin J Sport Med.

[2] Prodromidis AD, Drosatou C, Thivaios GC, Zreik N, Charalambous CP (2021). Timing of anterior cruciate ligament reconstruction and relationship with meniscal tears: a systematic review and Meta-analysis. Am J Sports Med.

[3] Montalvo AM, Schneider DK, Silva PL, Yut L, Webster KE, Riley MA, Kiefer AW, Doherty-Restrepo JL, Myer GD (2019). “What’s my risk of sustaining an ACL injury while playing football (soccer)?” a systematic review with meta-analysis. Br J Sports Med.

[4] Ueno R, Navacchia A, Bates NA, Schilaty ND, Krych AJ, Hewett TE (2020). Analysis of internal knee forces allows for the prediction of rupture events in a clinically relevant model of anterior cruciate ligament injuries. Orthop J Sports Med.

[5] Takahashi S, Nagano Y, Ito W, Kido Y, Okuwaki T (2019). A retrospective study of mechanisms of anterior cruciate ligament injuries in high school basketball, handball, judo, soccer, and volleyball. Medicine (Baltimore).

[6] Cannon J, Cambridge EDJ, McGill SM (2019). Anterior cruciate ligament injury mechanisms and the kinetic chain linkage: the effect of proximal joint stiffness on distal knee control during bilateral landings. J Orthop Sports Phys Ther.

[7] Cibulka MT, Bennett J (2020). How weakness of the tensor fascia lata and gluteus maximus may contribute to ACL injury: a new theory. Physiother Theory Pract.

[8] Homan KJ, Norcross MF, Goerger BM, Prentice WE, Blackburn JT (2013). The influence of hip strength on gluteal activity and lower extremity kinematics. J Electromyogr Kinesiol.

[9] Lessi GC, Serrão FV (2017). Effects of fatigue on lower limb, pelvis and trunk kinematics and lower limb muscle activity during single-leg landing after anterior cruciate ligament reconstruction. Knee Surg Sports Traumatol Arthrosc.

[10] Patrek MF, Kernozek TW, Willson JD, Wright GA, Doberstein ST (2011). Hip-abductor fatigue and single-leg landing mechanics in women athletes. J Athl Train.

[11] Dashti Rostami K, Naderi A, Thomas A (2019). Hip abductor and adductor muscles activity patterns during landing after anterior cruciate ligament injury. J Sport Rehabil.

[12] Willson JD, Petrowitz I, Butler RJ, Kernozek TW (2012). Male and female gluteal muscle activity and lower extremity kinematics during running. Clin Biomech Bristol Avon.

[13] Griffin LY, Agel J, Albohm MJ, Arendt EA, Dick RW, Garrett WE, Garrick JG, Hewett TE, Huston L, Ireland ML, Johnson RJ, Kibler WB, Lephart S, Lewis JL, Lindenfeld TN, Mandelbaum BR, Marchak P, Teitz CC, Wojtys EM (2000). Noncontact anterior cruciate ligament injuries: risk factors and prevention strategies. J Am Acad Orthop Surg.

[14] Powers CM (2010). The influence of abnormal hip mechanics on knee injury: a biomechanical perspective. J Orthop Sports Phys Ther.

[15] Ortiz A, Olson S, Trudelle-Jackson E, Rosario M, Venegas HL (2011). Landing mechanics during side hopping and crossover hopping maneuvers in noninjured women and women with anterior cruciate ligament reconstruction. PM R.

[16] Wang L-I (2011). The lower extremity biomechanics of single- and double-leg stop-jump tasks. J Sports Sci Med.

[17] Ueno R, Navacchia A, DiCesare CA, Ford KR, Myer GD, Ishida T, Tohyama H, Hewett TE (2020). Knee abduction moment is predicted by lower gluteus medius force and larger vertical and lateral ground reaction forces during drop vertical jump in female athletes. J Biomech.

[18] Husted RS, Bencke J, Hölmich P, Andersen LL, Thorborg K, Bandholm T, Gliese B, Lauridsen HB, Myklebust G, Aagaard P, Zebis MK (2018). Maximal hip and knee muscle strength are not related to neuromuscular pre-activity during sidecutting maneuver: a cross-sectional study. Int J Sports Phys Ther.

[19] Munn Z, Peters MDJ, Stern C, Tufanaru C McArthur A, Aromataris E. (2018) Systematic review or scoping review? Guidance for authors when choosing between a systematic or scoping review approach. BMC Med Res Methodol 1471-2288 10.1186/s12874-018-0611-x PMC6245623 30453902

[20] Peters MDJ, Godfrey CM, Khalil H, McInerney P, Parker D, Soares CB (2015). Guidance for conducting systematic scoping reviews. Int J Evid Based Healthc.

[21] Llurda-Almuzara L, Pérez-Bellmunt A, López-de-Celis C, Aiguadé R, Seijas R, Casasayas-Cos O, Labata-Lezaun N, Alvarez P (2020). Normative data and correlation between dynamic knee valgus and neuromuscular response among healthy active males: a cross-sectional study. Sci Rep.

[22] Cronin B, Johnson ST, Chang E, Pollard CD, Norcross MF (2016). Greater hip extension but not hip abduction explosive strength is associated with lesser hip adduction and knee Valgus motion during a single-leg jump-cut. Orthop J Sports Med.

[23] Smeets A, Vanrenterghem J, Staes F, Vandenneucker H, Claes S, Verschueren S (2020). Are anterior cruciate ligament-reconstructed athletes more vulnerable to fatigue than uninjured athletes?. Med Sci Sports Exerc.

[24] Hogg JA, Ackerman T, Nguyen A-D, Ross SE, Schmitz RJ, Vanrenterghem J, Shultz SJ (2021). The effects of gluteal strength and activation on the relationship between femoral alignment and functional Valgus collapse during a single-leg landing. J Sport Rehabil.

[25] Dai B, Heinbaugh EM, Ning X, Zhu Q (2014). A resistance band increased internal hip abduction moments and gluteus medius activation during pre-landing and early-landing. J Biomech.

[26] Rath ME, Stearne DJ, Walker CR, Cox JC (2016). Effect of foot type on knee valgus, ground reaction force, and hip muscle activation in female soccer players. J Sports Med Phys Fitness.

[27] Sinsurin K, Valldecabres R, Richards J (2020). An exploration of the differences in hip strength, gluteus medius activity, and trunk, pelvis, and lower-limb biomechanics during different functional tasks. Int Biomech.

[28] Lubahn AJ, Kernozek TW, Tyson TL, Merkitch KW, Reutemann P, Chestnut JM (2011). Hip muscle activation and knee frontal plane motion during weight bearing therapeutic exercises. Int J Sports Phys Ther.

[29] Russell KA, Palmieri RM, Zinder SM, Ingersoll CD (2006). Sex differences in valgus knee angle during a single-leg drop jump. J Athl Train.

[30] Hollman JH, Galardi CM, Lin I-H, Voth BC, Whitmarsh CL (2014). Frontal and transverse plane hip kinematics and gluteus maximus recruitment correlate with frontal plane knee kinematics during single-leg squat tests in women. Clin Biomech Bristol Avon.

[31] Nakagawa TH, Moriya ETU, Maciel CD, Serrão FV (2012). Trunk, pelvis, hip, and knee kinematics, hip strength, and gluteal muscle activation during a single-leg squat in males and females with and without patellofemoral pain syndrome. J Orthop Sports Phys Ther.

[32] Sritharan P, Lin Y-C, Pandy MG (2012). Muscles that do not cross the knee contribute to the knee adduction moment and tibiofemoral compartment loading during gait. J Orthop Res.

[33] Pohl MB, Kendall KD, Patel C, Wiley JP, Emery C, Ferber R (2015). Experimentally reduced hip-abductor muscle strength and frontal-plane biomechanics during walking. J Athl Train.

[34] McLean SG, Oh YK, Palmer ML, Lucey SM, Lucarelli DG, Ashton-Miller JA, Wojtys EM (2011). The relationship between anterior tibial acceleration, tibial slope, and ACL strain during a simulated jump landing task. J Bone Joint Surg Am.

[35] Henriksen M, Aaboe J, Simonsen EB, Alkjaer T, Bliddal H (2009). Experimentally reduced hip abductor function during walking: implications for knee joint loads. J Biomech.

[36] Kim D, Unger J, Lanovaz JL, Oates AR (2016). The relationship of anticipatory gluteus Medius activity to pelvic and knee stability in the transition to single-leg stance. PM R.

[37] Padua DA, Bell DR, Clark MA (2012). Neuromuscular characteristics of individuals displaying excessive medial knee displacement. J Athl Train.

[38] Fadaei Dehcheshmeh P, Gandomi F, Maffulli N (2021). Effect of lumbopelvic control on landing mechanics and lower extremity muscles’ activities in female professional athletes: implications for injury prevention. BMC Sports Sci Med Rehabil.

[39] Neamatallah Z, Herrington L, Jones R (2020). An investigation into the role of gluteal muscle strength and EMG activity in controlling HIP and knee motion during landing tasks. Phys Ther Sport.

[40] Baker RL, Souza RB, Rauh MJ, Fredericson M, Rosenthal MD (2018). Differences in knee and hip adduction and hip muscle activation in runners with and without iliotibial band syndrome. PM & R: the journal of injury, function, and rehabilitation.

[41] DeJong AF, Mangum LC, Resch JE, Saliba SA (2019). Detection of gluteal changes using ultrasound imaging during phases of gait in individuals with medial knee displacement. J Sport Rehabil.

[42] Stearns-Reider KM, Straub RK, Powers CM (2021) Hip abductor rate of torque development as opposed to isometric strength predicts peak knee Valgus during landing: implications for anterior cruciate ligament injury. J Appl Biomech:1–6 10.1123/jab.2020-0398 34544900

[43] Sharifi M, Shirazi-Adl A, Marouane H (2020). Sensitivity of the knee joint response, muscle forces and stability to variations in gait kinematics-kinetics. J Biomech.

[44] Nguyen A-D, Shultz SJ, Schmitz RJ, Luecht RM, Perrin DH (2011). A preliminary multifactorial approach describing the relationships among lower extremity alignment, hip muscle activation, and lower extremity joint excursion. J Athl Train.

